# A novel root canal filling technique using a resin-based sealer without gutta-percha: a summary of two cases

**DOI:** 10.1186/s12903-025-06957-5

**Published:** 2025-10-08

**Authors:** Motoki Okamoto, Kiichi Moriyama, Nanako Kuriki, Yusuke Takahashi, Julian G. Leprince, Maiko Suzuki, Koji Iwasaki, Henry Fergus Duncan, Mikako Hayashi

**Affiliations:** 1 Department of Oral Science and Translational Research, College of Dental Medicine, Nova Southeastern University, Fort Lauderdale, FL USA; 2 Department of Restorative Dentistry and Endodontology, Osaka University Graduate School of Dentistry, Suita, Osaka Japan; 3 Division of Cariology & Endodontology, University Clinics of Dental Medicine, Geneva, Switzerland; 4 Academic Clinical Research Center, Department of Medical Innovation, Osaka University Hospital, Suita, Osaka Japan; 5 Division of Restorative Dentistry and Periodontology, Trinity College Dublin, Dublin Dental University Hospital, Dublin, Ireland

**Keywords:** Resin-based sealer, Methacrylic resin, Root canal sealer, Root canal filling, Case series, Outcome

## Abstract

**Objectives:**

Root canal filling is a fundamental step in non-surgical root canal treatment (RCT), required to prevent reinfection or new infection within the root canal system. Traditionally root canal filling uses a sealer in combination with gutta-percha (GP). Here, two cases are presented in which the root canals were filled using only a methacrylic resin-based sealer without GP.

**Materials and methods:**

Two patients (one male, one female) required RCT – one due to irreversible pulpitis and the other to facilitate a subsequent post-retained protheses. Each RCT was performed according to a standardized procedure, before root canal filling using only methacrylic resin sealer.

**Results:**

Fillings were completed in curved and straight root canals without evidence of apical extrusion. No clinical symptoms or signs of dissolution were observed during follow-up (24 and 12 months in the respective cases).

**Conclusions:**

Our findings demonstrate the potential applicability of this novel technique for root canal filling using only a methacrylic resin-based sealer without GP.

**Clinical relevance:**

The success of our RCT cases offers evidence to support further clinical studies that compare the outcomes with standard root canal procedures.

**Supplementary Information:**

The online version contains supplementary material available at 10.1186/s12903-025-06957-5.

## Introduction

Root canal filling (RCF) is performed after a sterile environment is achieved during root canal treatment (RCT). Although the primary purpose of RCF is to prevent new infection or reinfection, it plays a secondary role in “entombing” residual bacteria. Traditionally, RCF employs lateral and vertical condensation techniques with gutta-percha (GP) [1], establishing this material as the primary component of the RCF core and minimizing the amount of root canal sealer. However, recent advancements in root canal sealers, including the development of hydraulic calcium silicate-based and bioceramic sealers, have popularized single-cone, “cold” techniques [2]. These methods use tapered cones of GP that increase the amount of sealer from conventional levels. Clinical studies evaluating this approach have found that outcomes were equivalent to traditional lateral condensation [3]. This is attributable to the improved mechanical properties of modern sealers, including enhanced wettability and minimal shrinkage during setting. Specifically, the superior clinical performance of hydraulic calcium silicate-based and bioceramic sealers is the result of better sealing due to calcium phosphate precipitation [4]. Because GP lacks high sealing ability and biocompatibility, this technological advancement represents a shift from GP-based RCFs to root canal sealers as the primary obturating material.

MetaSEAL soft paste (Sun Medical, Shiga, Japan) is a recently introduced methacrylic resin-based sealer available only in Japan. The material has been proposed as an alternative to hydraulic calcium silicate-based and bioceramic sealers in non-surgical, single-point RCT. MetaSEAL soft paste chemically cures in the root canal even in the presence of residual moisture, but still remains pliable, allowing for easy removal with hand or NiTi files. This pliability is attributed to the reducing effect of hydrophilic amino acid-based polymerization initiators. Furthermore, laboratory studies have demonstrated that MetaSEAL (used alone) forms tags in dentinal tubules, contributing to its sealing ability, which worsens when combined with GP [5]. The methacrylic resin sealer also micro-expands after absorbing moisture from subcutaneous tissues, further improving its sealing ability after RCF [6]. Animal experiments investigating adverse effects of the resin component, including its role in prolonging inflammation and polymerization shrinkage, found no abnormal inflammatory reactions; indeed, the inflammatory response was comparable to that of fatty acid-based zinc oxide and bioglass-containing sealers [6].

Based on these preliminary findings, we propose that RCF without GP is a promising next-generation technique. In support, we present a novel proof-of-principle study involving two cases that filled root canals with methacrylic resin sealer alone.

## Case report

The cases are described in accordance with the CARE guidelines (checklist in Supplementary Material 1). The two cases were part of a prospective observational study demonstrating the effectiveness of root canal obturation using a methacrylic resin sealer without GP, approved by the Ethics Committee of the Graduate School of Dentistry, Osaka University (number R3-E17). Treatments were performed by an endodontist with more than 10 years of experience and were in accordance with the Declaration of Helsinki. Informed consent has been obtained from the patient for publication as a case report of the process of treatment including clinical images.

### Case 1: successful methacrylic resin-based root Canal sealer obturation without GP

This case highlights the effectiveness of GP-free root canal filling using a methacrylic resin-based sealer. The patient timeline is presented in Table 1.

**Table 1:**
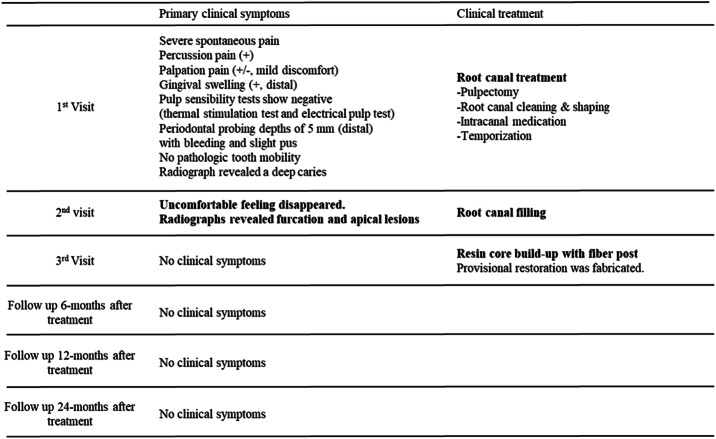
Timeline symptoms and procedures performed at dental hospital -Case 1

**Fig. 1:**
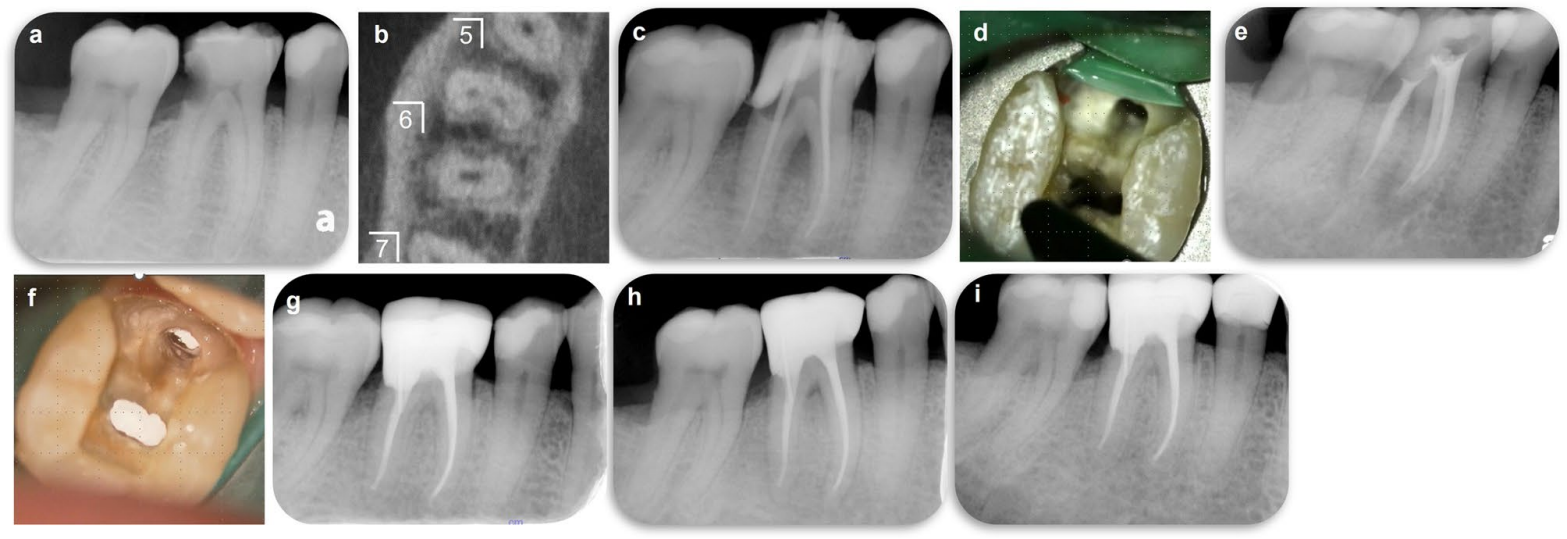
Case 1: Successful methacrylic resin-based root canal sealer obturation without gutta-percha. (**a**) Pre-operative radiograph; (**b**) pre-operative cone-beam computed tomography image; (**c**) pre-operative radiograph confirming the working length and demonstrating furcation lesions in the tooth; (**d**) image capture from a video recorded using a DOM demonstrating the resin-based sealer obturation procedure performed without gutta-percha; (**e**) post-operative radiograph immediately post-root canal obturation; (**f**) image captured from a video recorded using a dental operating microscope before the resin core build-up procedure, involving the placement of an optical fiber post; and (**g**–**i**) follow-up radiographs taken at 6 months, 1 year, and 2 years post-obturation, demonstrating long-term success

A 75-year-old Asian male with a history of smoking attended with multiple carious lesions. The mandibular first right molar had dental caries; however, the patient had no clinical symptoms. Travel restrictions during the coronavirus disease (COVID-19) pandemic prevented him from visiting the dentist. The patient experienced temperature-induced (cold water) pain in his lower right jaw and attended hospital 2 days later with severe pain. No facial asymmetry or swelling was observed; however, the affected tooth exhibited spontaneous pain with sensitivity to percussion, mild discomfort from palpation, distal gingival swelling, but no mobility (0 degrees). The distal aspect probing depth was 5 mm. Pulp sensibility testing showed no response to electrical (Digitest II; MORITA, Kyoto, Japan) or thermal cold (Pulper; GC, Tokyo, Japan) stimulation. The patient had no clinically relevant medical or family history, and no history of orthodontia, parafunctional habits, abnormal occlusal patterns, or dentures/implants. Oral hygiene was well-maintained. Adjacent teeth responded positively to pulp sensibility tests. A pre-operative radiography revealed a carious lesion extending distally to the pulp (Fig. 1a), although no radiolucency was observed at the apex of the lower right first molar. Previous cone-beam computed tomography demonstrated three root canals, one distal and two mesial, with no obvious abnormalities (Fig. 1b).

The patient was diagnosed with irreversible pulpitis and symptomatic apical periodontitis in the right mandibular first molar. Pulpotomy was recommended as the initial treatment, and the patient was informed that potential distal tooth structure loss might necessitate a crown-lengthening procedure prior to endodontic or prosthetic treatment. Tooth extraction and placement of a bridge or dental implant was discussed as another treatment option.

Following the pre-operative clinical evaluation, treatment was performed under magnification using a dental operating microscope ([DOM], M320-D; Leica Microsystems, Wetzlar, Germany). Rubber dam isolation was performed under local anesthesia and the infected distal dentin was removed using a diamond bur with a water-cooled high-speed handpiece and manual excavator, resulting in exposure into the pulp chamber. The distal root pulp tissue appeared necrotic, although the mesial root exhibited significant bleeding, and the patient reported pain during instrumentation. Necrosis led to the choice of an RCT over a pulpotomy.

Apical patency was maintained using a #6 hand file (Ready Steel C Plus; Dentsply Sirona, Ballaigues, Switzerland), and the root canal length was measured electronically using an apex locator (Root ZX3; MORITA, Kyoto, Japan). A glide path was established, and the root canal was prepared using a NiTi file system (HyFlex EDM; Coltene-Whaledent, Altstätten, Switzerland) with an endodontic motor (TriAuto ZX2; MORITA), allowing for simultaneous electronic root canal length measurements. The root canal was thoroughly irrigated with 2.5% sodium hypochlorite solution (Neo Cleaner; Neo Dental Chemical Products Co., LTD, Tokyo, Japan) and 3% ethylenediaminetetraacetic acid solution (Smear Clean; Nishika, Yamaguchi, Japan) using a 27-gauge irrigation syringe (Neo Dental Chemical Products Co.). Simultaneous agitation was performed using an NiTi instrument (X-p Endo Finisher #25; FKG Dentaire, La Chaux-de-Fonds, Switzerland). The canal was dried with sterile paper points and a non-setting calcium hydroxide dressing applied (Calcipex II; Nishika). A temporary glass ionomer cement was placed (Basecement; Shofu Inc, Kyoto, Japan).

Pain had subsided by the second visit, when the root canal length was measured. After the canal was shaped using a NiTi file and cleaned as described above, its working length was confirmed using periapical radiography with a GP cone (Fig. 1d). Canals were re-irrigated, dried (Fig. 1d), and then obturated using a methacrylic resin sealer (MetaSEAL soft paste; Sun Medical) without GP (Fig. 1e), which has a working time of approximately 40 s. An accompanying video was recorded with the DOM. Following RCF, prosthetic procedures, including placement of a resin core build-up with a fiber post, were performed.

Follow-up appointments were scheduled (Figs. 1g–i), allowing the tooth to be observed for 24 months (Table 1). Subsequent symptoms or abnormal clinical or radiographic findings were absent, demonstrating that the RCT was successful.

### Case 2: limitations of methacrylic resin-based root Canal sealer without GP

This case highlights certain limitations for this novel GP-free RCT technique and offers insight for improvement. The case timeline is presented in Table 2.

**Table 2:**
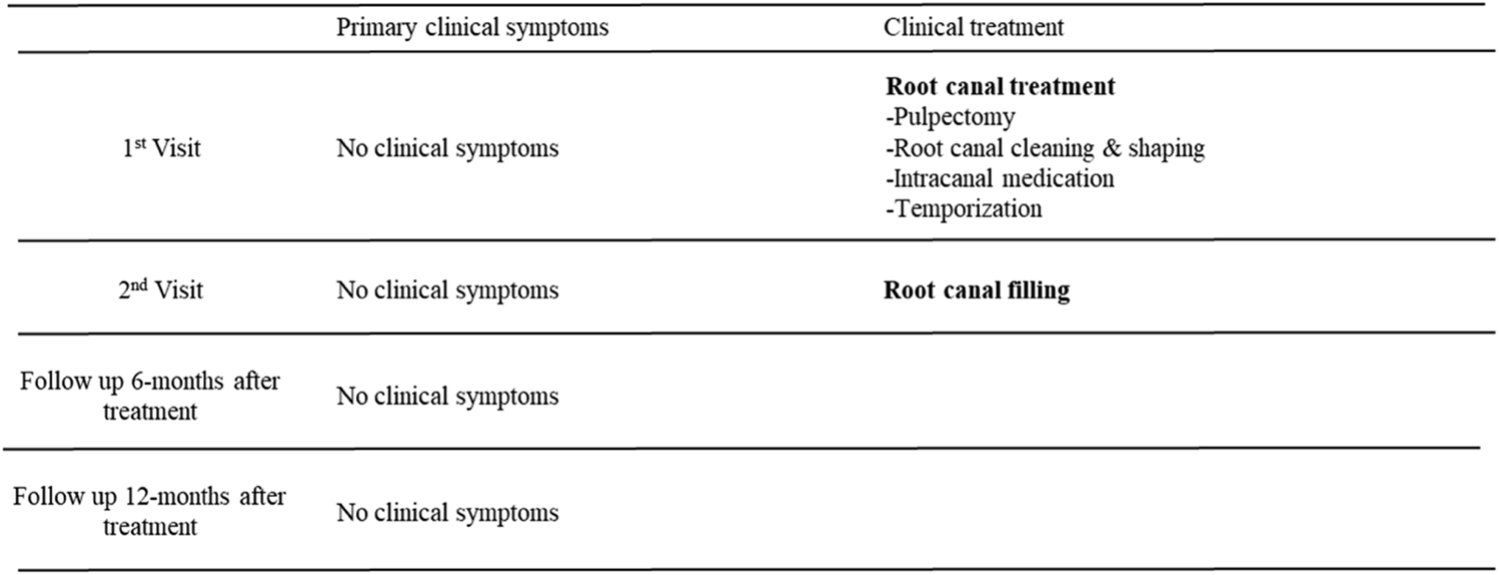
Timeline symptoms and procedures performed at dental hospital -Case 2

**Fig. 2:**
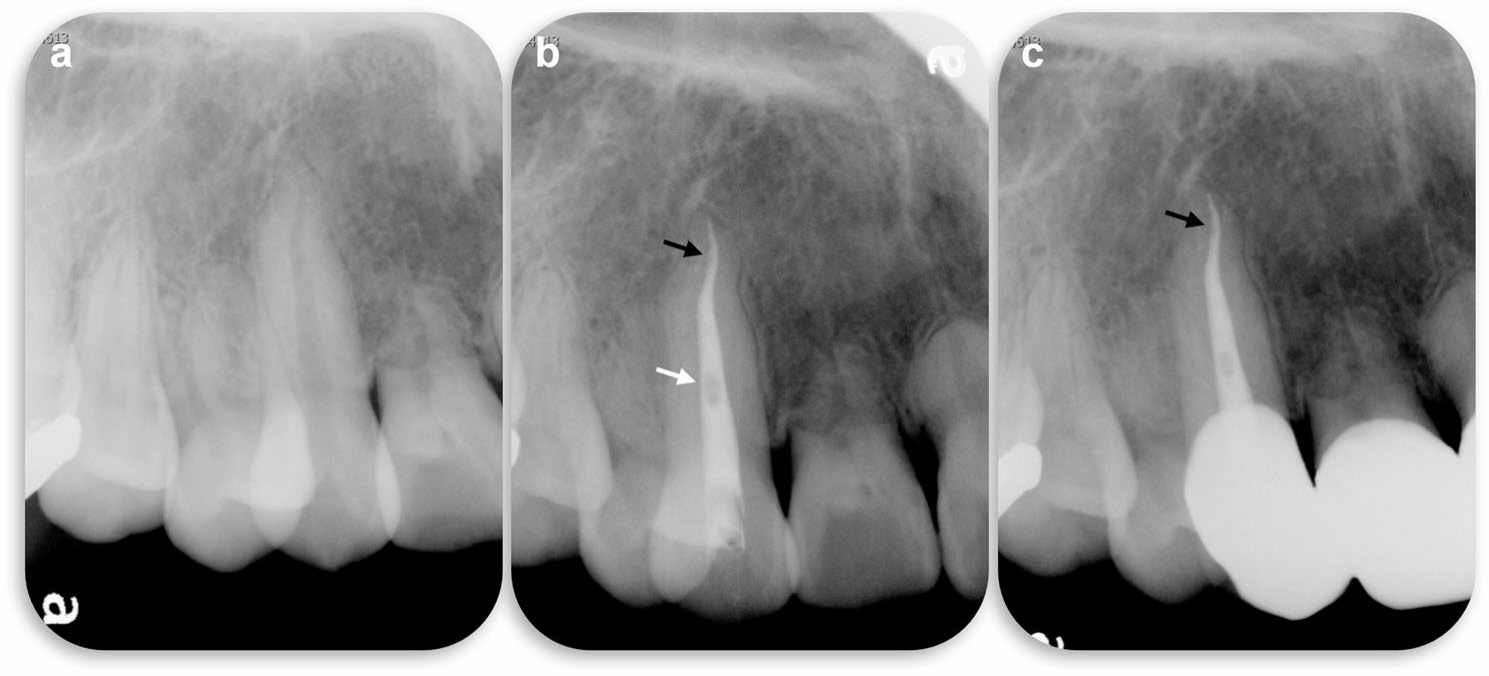
Case 2: Limitations and improvements of methacrylic resin-based root canal sealers without gutta-percha. (**a**) Pre-operative radiograph; (**b**) post-operative radiograph obtained immediately after RCF performed without gutta-percha showing a notable improvement with this obturation technique with the presence of only a small void (black arrow), while larger voids in the center of the canal (white arrow) may improve as prosthodontist gain more experience with techniques that do not rely on gutta-percha; (**c**) follow-up radiograph obtained 1 year after treatment showing no loss of sealer (black arrow)

A 24-year-old Asian woman with no smoking history was referred from a private clinic to the University clinic due to root resorption of the maxillary lateral and central incisors. She had a history of orthodontic treatment. The prosthodontics department developed a treatment plan that included RCT of the maxillary right canine to facilitate subsequent restoration. The patient had no systemic disease or any clinical symptoms. Pre-operative radiography revealed no pathology associated with the canines; however, shortening of the lateral incisor roots was observed, and the canine root exhibited an S-shape morphology (Fig. 2a). This case report focuses on the treatment of the right maxillary canine, although the left canine was also treated.

The maxillary anterior region was isolated using a rubber dam under local anesthesia. All procedures were performed using a DOM (M320-D; Leica). The RCT was initiated through the incisal surface using a diamond bur with a water-cooled high-speed handpiece. As in Case 1, apical patency was maintained using a #6 hand file, and the working length was determined using an apex locator. Canals were prepared as described in Case 1. Both canines received the same treatment and were temporarily sealed with glass ionomer cement. At the second visit, the working length was confirmed via radiography (Fig. 2b), and the root canal was filled with a methacrylic resin sealer (MetaSEAL soft paste; Sun Medical) without GP, again as in case 1.

Subsequently, the patient was referred to a prosthodontist for placement of a fixed bridge restoration. No apical radiolucency was detected at the 1-year follow-up (Fig. 2c).

This RCF exhibited voids that highlight an area for potential improvement, despite not affecting the clinical outcome. The relatively large void observed in the center of the root canal (Fig. 2b, white arrow) is likely attributable to miscommunication during the procedure. Originally, the void would have been removed during post-preparation, but because a sufficient ferrule effect was achieved, post-preparation was minimized and the void remained unaddressed.

Void presence highlights the challenges in predictable sealer placement and the importance of careful delivery. Another limitation is the micro void at the root apex (Fig. 2b, black arrow). This finding can also be observed in single-point RCFs with high sealer content. Despite this potential limitation, we did not observe any clinical signs or symptoms after 1 year. Therefore, the RCT was considered successful.

## Discussion

**Fig. 3:**
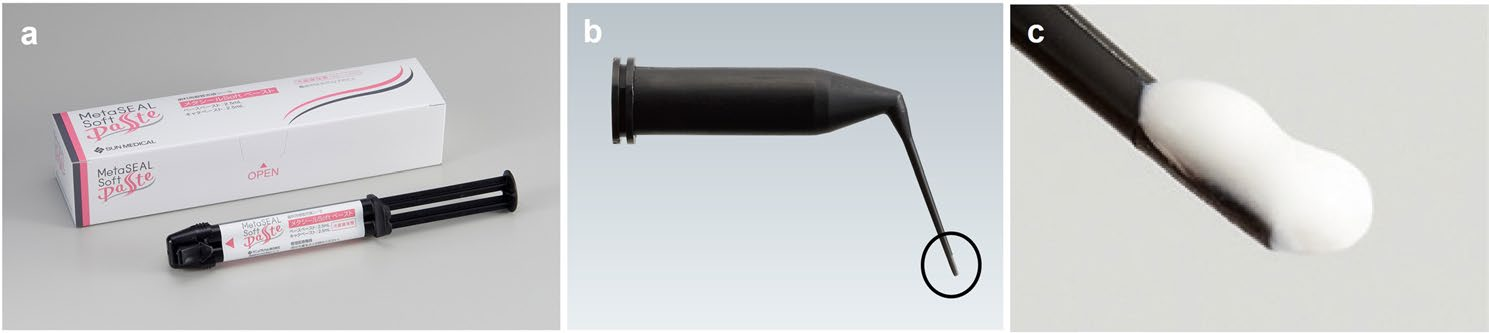
Methacrylic resin sealer and dedicated filling tip with side vent. (**a**) MetaSEAL soft paste available in Japan; (**b**) dedicated filling tip with side vent; (**d**) enlarged image of the black circle in panel B. The special tip is filled with sealer for insertion into the root canal

The reported cases demonstrate the successful use of a GP-free methacrylic resin root canal-filling sealer. Although further studies are needed to validate its potential as a large-scale alternative to conventional RCF methods, this case report provides foundational data for further clinical research and highlights the potential applications of this novel technique. Following our work, a randomized controlled trial (jRCTs052240103) has been initiated to compare the clinical outcomes of this technique and the conventional single-point technique with hydraulic calcium silicate-based sealer, as well as the vertical compaction technique with epoxy resin sealer. The methacrylic resin sealer used in this study possesses characteristics distinct from those of ‘Metaseal’ (Parkell Inc, Edgewood, NY, USA) and exhibits elastic properties after curing. An ideal root canal sealer should demonstrate the following properties described by Grossman: dimensional stability, impermeability to moisture, radiopacity, non-irritating to periapical tissues, antibacterial properties, ease of manipulation, and adhesion to canal walls [7]. Among these, the present sealer appears to meet al.l criteria except for antibacterial properties. Notably, the hydrophilic amino acid-based polymerization initiator used in this sealer is not inhibited by residual moisture on the root canal wall and is relatively unaffected by sodium hypochlorite solution. However, data regarding its biocompatibility—specifically cytotoxicity, allergenicity, and mutagenicity [8]—are currently limited, and further investigation is necessary to fully assess its material science and biological profile. The methacrylic resin sealer used in this study was prepared from mixing equal parts of base and catalyst to achieve an ideal consistency (Fig. 3a). When proper irrigation was performed, the sealer adapted well to the isthmuses and curved canals (Figs. 1e and 2b). As demonstrated in animal studies [6], the resin did not cause prolonged inflammation or postoperative pain. Methacrylic resin sealers do not precipitate structures containing calcium (Ca) or phosphorus (P) as bioceramic or bioglass-containing sealers do, which might be considered a disadvantage. However, comprehensive gene analysis using RNA sequencing has shown that methacrylic resin promotes wound healing in the sealer-applied environment and increases the expression of anti-inflammatory genes, making it a desirable material for root canal filling [6]. Additionally, despite the large volume and extensive contact area of the sealer, dissolution was minimal (Figs. 1g–i and 2b–c). However, as the follow-up period for these cases was relatively short (2 years), extended observation is essential [9]. Laboratory research has demonstrated that epoxy resin sealers have lower solubility than bioceramic sealers, suggesting that this methacrylic resin sealer should perform well [10]. Another notable feature of this treatment is a considerably shortened procedure time (40 s working time to fill three root canals), a significant advantage in endodontic treatments, which are typically time-consuming. This sealer has a working time of approximately 10 min in an indoor environment and can be handled in the same way as conventional sealers. Additionally, because this method relies solely on a root canal sealer and the use of a side vent tip (Fig. 3), it may be effective in resource-limited environments where access to specialized tools is restricted.

One potential limitation of this technique is potential void formation owing to the absence of a GP core. Previous studies have suggested that the presence of voids in root canal fillings may negatively affect clinical outcome [11]. Although these findings are based on techniques involving gutta-percha points, and thus cannot be directly compared with our sealer-only approach, the presence of voids—such as those observed in Case 2—should not be overlooked. Case 2 also highlights the risk of miscommunication between the endodontist and prosthodontist that can arise during the early implementation of this treatment method. Two types of voids were observed: a small apical micro-void (Fig. 2, black arrow) and a large, potentially correctable void in the centre of the root canal (Fig. 2, white arrow). Postoperative radiographs revealed large voids (Fig. 2B, white arrow) in the centre of the root canal filling. In this instance the prosthodontist was asked to remove the void and proceed with prosthetic treatment. However, based on the assessment that the remaining ferrule was sufficient and excessive preparation for resin build-up was unnecessary, the preparation was performed without magnification or radiographic confirmation. As a result, the void had inadvertently remained. Although removing the void was proposed, the patient did not consent to additional treatment due to the absence of clinical symptoms. Thus, prosthetic treatment was completed with the patient’s informed consent. To date, there have been no clinical signs of failure related to this void. Nevertheless, we acknowledge that such miscommunication may occur during the early stages of implementing this new gutta-percha-free root canal filling technique. This case highlights the importance of robust interdisciplinary communication. Small voids can also occur in single-point cold RCF techniques due to increased sealer content [12]. Given the aims of RCF, minimizing gaps is critical; auxiliary techniques such as ultrasonic activation may be a potential solution [13]. Although the presence of voids is an issue for our method, none of the available root canal filling techniques can completely eliminate voids [14], and they also do not exhibit significant clinical differences in this regard [15–17]. The ongoing randomized controlled trial is planned to solve these problems by using small sealer tip (30) and counter-clockwise rotation with a NiTi file. Currently, we cannot determine whether voids in resin-based RCF without CP (Figs. 2b–c) negatively affect the prognosis of endodontic treatment. According to established clinical guidelines, a minimum follow-up period of one year—and ideally as long as possible—is recommended to evaluate the outcomes of root canal treatment [18]. In the present study, the follow-up periods were 2 years and 1 year, respectively. Although the minimum recommended duration has been met, we acknowledge that regular and long-term observation remains important to fully assess treatment success. (i.e., our clinical trial: jRCTs052240103). Furthermore, the available data on the methacrylic resin-based sealer used in this study remain limited. In microleakage tests using glucose as a tracer, methacrylic resin-based sealers such as Resilon and Epiphany have not demonstrated superior sealing performance compared to the epoxy resin-based sealer AH Plus [19]. However, a recent study reported that Metaseal Soft Paste exhibited improved sealing ability over AH Plus [5]. At present, it remains unclear whether these differences are attributable to advancements in material composition or variations in testing methodologies. Further investigation is needed, including addressing the biocompatibility concerns mentioned above. The most significant feature in these cases was the absence of GP. Although traditionally utilized as the core material for root canal obturation, GP lacks adhesive and sealing properties within the root canal, leaving doubts as to its long-term reliability. Hence, GP is increasingly being replaced by modern sealers, such as the resin-based sealer used here. Obturation using such sealers improve prosthodontic adhesion, a considerable benefit given that RCF quality and coronal restoration significantly influence the prognosis of endodontic treatment [20]. Additionally, the resin appears to be readily removable in the event of recurrent disease. Preliminary studies using transparent root canal model and extracted human teeth have confirmed that the resin can be extracted with hand files, NiTi files, and ultrasonically assisted file-shaped tips (Unpublished data). Another concern is the predictability of sealer placement and its extent within the root canal. In these cases, a side-vented tip was used to minimize apical pressure and prevent sealer extrusion beyond the root apex (Fig. 3b, c). Prior to clinical application, we confirmed sealer flow using a transparent root canal model on a benchtop. Preliminary training indicated that this technique was quickly mastered. The cases presented were initial treatments and no large periapical lesions were observed preoperatively. Future clinical studies will be needed to determine whether favorable outcomes can also be achieved in cases involving large periapical lesions or root canal systems that have been severely compromised or complicated.

To the best of our knowledge, this report is the first to empirically highlight the effectiveness and applicability of a novel RCF method using a methacrylic resin sealer without GP. Unlike conventional obturation techniques, our approach saves time and does not require specialized instruments. Although the demonstration of its efficacy was limited to two successful cases, the promising outcomes provide impetus for further clinical studies that could broaden the global applicability of MetaSEAL soft paste.

## Supplementary Information

Supplementary Material 1.

Supplementary Material 2.

Supplementary Material 3.

## Data Availability

Data is provided within the manuscript or supplementary information files.

## Abbreviations

RCT: Root canal treatment
GP: Gutta-percha
RCF: Root canal filling
DOM: Dental operating microscope
Ca: Calcium
P: Phosphorus
